# A comparative study of general and severe mycoplasma pneumoniae pneumonia in children

**DOI:** 10.1186/s12879-024-09340-x

**Published:** 2024-04-26

**Authors:** Shuo Yang, Sukun Lu, Yinghui Guo, Wenjun Luan, Jianhua Liu, Le Wang

**Affiliations:** 1 Institute of Pediatric Research, Children’s Hospital of Hebei, 133 Jianhua South Street, Shijiazhuang, 050031 China; 2 Department of Respiratory, Children’s Hospital of Hebei, Shijiazhuang, 050031 China; 3 Department of Laboratory Medicine, Children’s Hospital of Hebei, Shijiazhuang, 050031 China; 4 Children’s Hospital of Hebei, Shijiazhuang, 050031 China

**Keywords:** Disease severity, Children, *Mycoplasma pneumoniae* pneumonia

## Abstract

**Objectives:**

The increasing prevalence of severe *Mycoplasma pneumoniae* pneumonia (SMPP) poses a significant threat to the health of children. This study aimed to characterise and assess the outcomes in children with SMPP.

**Methods:**

We retrospectively analysed children hospitalised for *M. pneumoniae* pneumonia (MPP) between January and December 2022. Retrospectively, demographic, clinical, underlying diseases, laboratory and radiological findings, and treatment outcomes were collected and analysed. Disease severity was defined as severe or general according to the Guideline for diagnosis and treatment of community-acquired pneumonia in children (2019 version).

**Results:**

Over a 12-month observation period, 417 children with MPP were enrolled, 50.6% (211/417) of whom had SMPP, with the peak incidence observed in winter. Of the 211 children with SMPP, 210 were treated and discharged with improvement, while one child with congenital heart disease died of cardioembolic stroke. A significantly higher proportion of patients with SMPP had underlying diseases, extrapulmonary complications (myocardial and digestive system involvement), and bacterial co-infection. A total of 25 (12%) children with SMPP received mechanical ventilation. The median duration of mechanical ventilation was 3 days. All children were treated with macrolide antibiotic. A significantly higher proportion of patients with SMPP received antibiotic other than macrolides, methylprednisolone sodium succinate, intravenous immunoglobulin and anticoagulation, compared with patients with general MPP (GMPP). Children with SMPP had significantly higher levels of white blood cells, neutrophil percentage, C-reactive protein, procalcitonin, interferon-γ, interleukin (IL)-2, IL-5, IL-6, IL-8, IL-10 and significantly lower percentages of lymphocytes, monocytes, and natural killer cells, compared with GMPP group.

**Conclusion:**

Our findings suggest that severely ill children have more pronounced inflammatory reaction and extrapulmonary complications. For effective management of children with SMPP, hormonal, prophylactic, anticoagulant therapy, as well as the use of antibiotics other than macrolides for bacterial co-infections, could be incorporated into treatment regimens.

## Background

Severe pneumonia is the leading infectious disease that causes death in children under five years of age. It is estimated that 740,180 deaths occur annually worldwide due to severe pneumonia, accounting for 22% of deaths in children under 5 years of age [1]. *Mycoplasma pneumoniae* has long been considered an important aetiology of pneumonia and is commonly isolated from children [2]. Although most children with *M. pneumoniae* pneumonia (MPP) appreciably recover, some present with worsening clinical symptoms and imaging findings, defined as severe MPP (SMPP) [3]. In 2022, MPP was detected in most of children with pneumonia and caused a substantial increase in hospitalisations, severe illnesses, even deaths [4]. Research on active surveillance of hospitalised children suffering from SMPP in China is scarce [5, 6]. In order to improve treatment and to reduce morbidity and mortality, it is vital to recognize the factors contributing to the severity of MPP [7]. This study sought to characterise the demographic and clinical features of paediatric SMPP patients in 2022, which has been rarely described in the literature.

## Methods and materials

### Ethics approval

This study was carried out according to the protocol which was reviewed and approved by the Institutional Review Board of the Children’s Hospital of Hebei (CHH) (Approval No. 202101). The Ethics Committee of CHH approved this study protocol and waived the obligation for informed consent because of the retrospective nature of the study.

### Study participants

Paediatric patients (aged <16 years) admitted to our hospital between January and December 2022 with a discharge diagnosis of MPP were enrolled in this study. Demographics, clinical data, laboratory findings, radiological and bronchoalveolar lavage findings were retrieved from the inpatient electronic records and analysed retrospectively.

### MPP diagnoses and disease severity

MPP was confirmed according to Guideline for diagnosis and treatment of community-acquired pneumonia in children (2019 version) [8], including: 1) a presence of an infiltrate on chest radiography reported by two licensed radiologists, 2) fever, cough, or abnormal lung auscultation; and 3) the positive detection of *M. pneumoniae* DNA in lower respiratory tract specimens by real-time polymerase chain reaction (PCR) and the positive detection of specific MP antibody in sera using a micro-particle agglutination test. According to the Chinese guidelines on CAP, severe disease was defined as the presence of one or more of the following manifestations: (1) Radiography: infiltration of 2/3 of one lung, multilobar infiltration, pleural effusion, pneumothorax, atelectasis, lung necrosis or lung abscesses; (2) Hypoxemia: cyanosis; marked increase in respiratory rate; marked chest wall retractions, tracheal tugging or nasal flaring; O_2_ saturation less than 92%; (3) Extrapulmonary complications; (6) Persistent high fever for more than 5 days; or (7) reluctance or inability to feed. All the other subjects were considered to have GMPP [9].

The exclusion criteria were as follows: 1) solid tumor or hematological malignancy 2) history of recent hospitalisation (<90 days), and 3) immune deficiency or received corticosteroids prior to admission. Cardiovascular complications included myocardial damage, pericardial effusion, and heart failure. Neurological involvement included febrile convulsions, epilepsy, intracranial haemorrhage, and encephalitis. Digestive complications included abnormal liver function, gastritis, and diarrhea.

### Pathogen detection

A multiplex PCR-based platform (GenomeLab system) was utilized to detect *M. pneumoniae* and ten other pathogens including influenza virus, respiratory syncytial virus, adenovirus, parainfluenza virus, rhinovirus, metapneumovirus, bocavirus, coronavirus and *Chlamydia pneumoniae*. Multiplex-PCR was performed as previously described [10]. Bacterial and fungal cultures of respiratory secretions and blood samples were obtained according to the protocols developed in our diagnostic laboratory. Specimens used for sputum culture included bronchoalveolar lavage fluid (BALF), negative pressure aspiration by tracheal intubation and induced sputum (IS). Children who underwent bronchial lavage were provided with bronchial lavage fluid. Children who underwent tracheal intubation provided the deep sputum by negative pressure suctioning through tracheal intubation. Other children provided induced sputum. A trained nurse utilized a sterile negative pressure suction catheter to induce a cough to obtain an IS sample. Evidence of bacterial co-infection in our study was demonstrated using blood cultures from sterile sites or induced sputum from non-sterile sites. For positive respiratory specimens, it was considered to be a bacterial co-infection if the clinician judged it clinically relevant and provided appropriate antibiotic treatment.

### Statistical analysis

Medians with interquartile ranges were used for continuous variables, and counts (%) were used for categorical variables. Continuous and categorical variables were analysed using the Mann–Whitney U test and Fisher’s exact test. Univariate analysis was performed to identify the differences between patients with SMPP and those with general MPP (GMPP). Logistic regression analysis was performed to select the variables associated with SMPP. Statistical significance was set at *p* < 0.05. 3. All data analyses were performed using SPSS (version 25.0; SPSS Inc., Chicago, IL, USA).

### Treatment and outcomes

All patients were treated according to the expert consensus on the diagnosis and management of *M. pneumoniae* in children [8]. Outcomes recorded were recovery, discharge, transfer to a community hospital, and death.

## Results

### General patients’ information

**Fig. 1:**
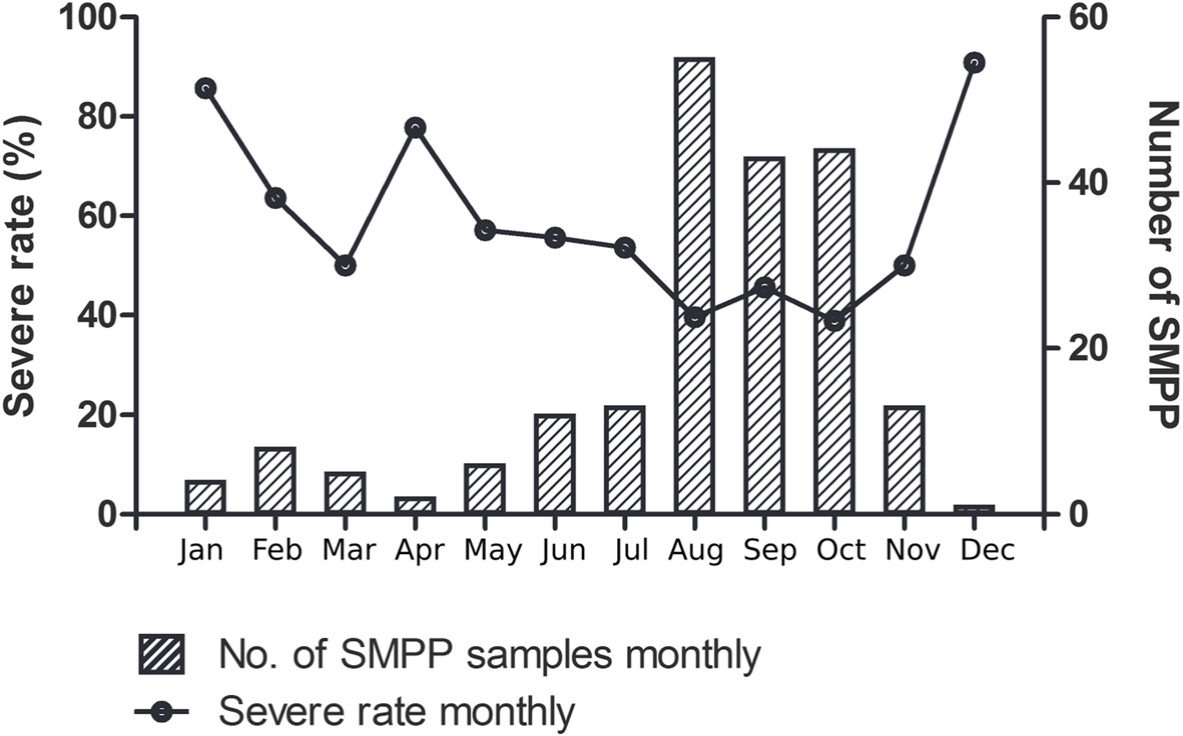
Severe MPP in children during 2022

During the observation period, 417 children with MPP were enrolled. The characteristics of the study cohort are presented in Table 1. The median age was 5.5 years, ranging from 0.2–15 years, with a female-to-male ratio of 0.75. The severe case rate was 50.6% (211/417). Analysis of the onset time showed that the epidemic occurred between August and October (autumn in the northern hemisphere) (Fig. 1). The peak period of SMPP was observed in winter. No significant differences in age or sex were found between the SMPP and GMPP groups (*p*>0.05; Table 1).

**Table 1:** Demographic, clinical features and outcomes of children with GMPP and SMPP

**Characteristics**		**GMPP (*n*=206)**	**SMPP (*n*=211)**	***P* value**
		n (%)	n (%)	
Demographics	Age (years)	6 (4, 8)	6 (3, 8)	0.213
	Gender (male/female)	86 (42%)	107 (51%)	0.066
Clinical features	Underlying disease	8 (3.9%)	29 (13.7%)	**<0.001**
	Disease day before admission	5.5 (3, 7)	7 (5, 10)	**<0.001**
	Febrile day before admission	2 (2, 3)	6 (2, 8)	**<0.001**
Extrapulmonary complications	Adenoid hypertrophy	4 (1.9%)	1 (0.5%)	0.211
	Acute otitis media	4 (1.9%)	5 (2.4%)	1.000
	Rhinosinusitis	10 (4.9%)	7 (3.3%)	0.428
	Cardiovascular system involvement	6 (2.9%)	16 (7.6%)	**0.033**
	Neurologic involvement	0 (0%)	5 (2.4%)	0.061
	Gastrointestinal involvement	12 (5.8%)	40 (19.0%)	**<0.001**
Treatment and outcomes	Antibiotic use^a^	70 (34%)	168 (79.6%)	**<0.001**
	Methylprednisolone sodium succinate	90 (43.7%)	173 (82%)	**<0.001**
	Intravenous immunoglobulin	0 (0%)	21 (10%)	**<0.001**
	Anticoagulation treatment	3 (1.5%)	36 (17.1%)	**<0.001**
	Mechanical ventilation use	0 (0%)	25 (12%)	**<0.001**
	Mechanical ventilation days	0	3 (2, 5)	**<0.001**
	Respiratory failure	0 (0%)	15 (7.1%)	**<0.001**
	Hospital duration	6 (5,8)	8 (7,12)	**0.007**
	Death	0 (0%)	1 (0.5%)	1.000

^a^Antibiotics other than macrolides

### Clinical presentation and outcome

The clinical features of the patients on admission are presented in Table 1. Compared to children with GMPP, those in the SMPP group had significantly longer fever and disease duration (both *p*<0.001) and hospital duration (*p*=0.007). A significantly greater proportion of patients in the SMPP group had underlying diseases (13.7% vs. 3.9%, *p*<0.001) and extrapulmonary complications, including myocardial (*p*=0.033) and gastrointestinal (*p*<0.001) involvement, than those in the GMPP group. A total of 25 RMPP cases warranted mechanical ventilation, with an median ventilation duration as 3 days (IQR 2, 5). The respiratory failure occurred in 15, with statistically significant differences compared to patients with GMPP (all *p*<.001, Table 1).

All children with GMPP either recovered or experienced an improvement, whereas one boy aged 7 years in the SMPP group died. He had underlying congenital heart disease. During admission, a cardioembolic stroke occurred, and a mucus plug was found under a fibreoptic bronchoscope.

### Treatment

All 417 children were treated with macrolide. A significantly higher proportion of patients with SMPP received antibiotic other than macrolides, methylprednisolone sodium succinate, intravenous immunoglobulin, and anticoagulant treatment (all *p*<0.001), compared with patients with GMPP (Table 1).

### Laboratory testing results

Laboratory tests showed that the SMPP group had a significantly greater percentage of white blood cells and neutrophils, and a significantly lower percentage of lymphocytes, monocytes, and natural killer (NK) cells (Table 2). In the SMPP group, the inflammatory indicators were remarkably greater including C-reactive protein (CRP), procalcitonin (PCT), as well as interferon (IFN)-γ, interleukin (IL)-2, IL-5, IL-6, IL-8, and IL-10 (Table 2). There were no significant differences in serum IL-4, IL-12p, IL-17A, IL-1β, IFN-α and tumor necrosis factor-α concentrations between the two groups (Table 2).

**Table 2:** Laboratory results of children with GMPP and SMPP infections

**Laboratory testing results**	**GMPP**	**SMPP**	***P* value**
	***n*=206**	***n*=211**	
WBC (10^9/L)	8.3 (6.9, 11)	10.3 (7.8, 14)	<0.001
Neutrophils%	57.7 (45.5, 65.3)	69.1 (58.3, 78.7)	<0.001
Lymphocytes%	33 (24.8, 43.9)	22.1 (14.2, 32.3)	<0.001
Monocytes%	7.9 (6.1, 25.2)	6.7 (4.7, 9.0)	<0.001
CRP (mg/L)	3.0 (0.8, 9.0)	13.7 (3.8, 36.3)	<0.001
PCT (ng/mL)	0.10 (0.07, 0.14)	0.14 (0.09, 0.30)	<0.001
CD3+ T cell%	73 (67, 77)	70 (63, 76)	0.124
CD4+ T cell%	41 (35, 46)	38 (31, 45)	0.070
CD8+ T cell%	24 (21, 28)	26 (21, 30)	0.192
CD19+ B cell%	17 (14, 21)	19 (14, 26)	0.053
Natural killer cell%	7.5 (5.1, 21.1)	6.4 (3.6, 10.0)	0.012
Interferon-γ (pg/mL)	5.7 (3.0, 9.0)	7.5 (3.9, 12.7)	<0.001
IL-2 (pg/mL)	1.5 (0.8, 2.8)	2.0 (1.0, 35)	0.016
IL-4 (pg/mL)	2.4 (1.5, 4.0)	2.6 (1.5, 4.3)	0.434
IL-5 (pg/mL)	1.2 (0.7, 2.0)	1.7 (0.9, 3.2)	<0.001
IL-6 (pg/mL)	10.9 (6.3, 17.4)	23.0 (10.7, 26.3)	<0.001
IL-8 (pg/mL)	7.7 (4.3, 12.0)	14.2 (7.5, 26.3)	<0.001
IL-10 (pg/mL)	4.2 (2.8, 6.1)	6.2 (3.8, 10.1)	<0.001
IL-12p70 (pg/mL)	3.7 (2.2, 6.5)	4.8 (2.6, 7.5)	0.059
IL-17A (pg/mL)	18.3 (14.2, 25.2)	18.5 (14.0, 25.8)	0.667
IL-1β (pg/mL)	3.7 (2.3, 6.9)	3.9 (2.1, 8.3)	0.411
Interferon-α (pg/mL)	4.6 (2.2, 11.3)	4.6 (2.3, 15.1)	0.341
TNF-α (pg/mL)	4.1 (2.7, 6.9)	4.4 (2.8, 7.9)	0.120

Values are presented as median (IQR)

*WBC* White blood cell, *TNF* Tumor necrosis factor, *IL* Interleukin, *CRP* C-reactive protein, *PCT* Procalcitonin

### Co-infections

Co-infections occurred more frequently in the SMPP group (80/211, 37.9%) than in the GMPP group (35/206, 17.0%) (*p*<0.001; Table 3). Among these, bacterial co-infections occurred in 25 SMPP (25/211, 11.8%) and 10 GMPP cases (10/206, 4.8%). The SMPP group had a significantly higher percentage of bacterial co-infections (*p*=.010; Table 3). Among these, *S. pneumoniae* was the most common bacterium, with 10 severe cases and three common cases. In addition, viral co-infections were observed in 25 and 55 patients in the GMPP and SMPP groups, respectively (*p*<0.001), with rhinovirus being the most frequently identified (Table 3). It should be noted that among the culture-positive strains, one case of *Staphylococcus epidermidis* and one case of *Staphylococcus capitis* were not treated with the appropriate antibiotics and were excluded as positive infections.

**Table 3:** Etiologic agents of children with GMPP and SMPP

**Etiological agents**	**Total**	**GMPP**	**SMPP**	***P* value**
	***n*=417**	***n*=206**	***n*=211**	
*M. pneumoniae* mono-detection	**302**	**171**	**131**	**<0.001**
*M. pneumoniae* co-detection	**115 (27.6%)**	**35 (17.0%)**	**80 (37.9%)**	
**Virus, n**	**80 (19.2%)**	**25 (12.1%)**	**55 (26.1%)**	**<0.001**
HRV	44	16	28	
HRV+HBoV	1	1	0	
HRV+PIV	2	1	1	
HRV+HMPV	1	1	0	
HRV+ADV	1	1	0	
RSV	9	3	6	
PIV	7	2	5	
2019-nCoV	5	0	5	
2019-nCoV+HRV	1	0	1	
HMPV	3	0	3	
HBoV	3	0	3	
ADV	2	0	2	
Influenza B	1	0	1	
**Bacteria, n**	**35 (8.4%)**	**10 (4.8%)**	**25 (11.8%)**	**0.010**
*S. pneumoniae*	8	2	6	
*S. pneumoniae+*HRV	2	1	1	
*S. pneumoniae+*HRV+ADV	1	0	1	
*S. pneumoniae+H. influenzae+*HRV	1	0	1	
*S. pneumoniae+S. aureus+*HRV	1	0	1	
*H. influenzae*	5	2	3	
*H. influenzae+*HRV	3	0	3	
*H. influenzae+*HBoV	1	0	1	
*S. aureus*	4	1	3	
*MTB*	2	1	1	
*P. aeruginosa+*HRV	2	1	1	
*M. catarrhalis*	1	1	0	
*M. catarrhalis+*HRV	1	1	0	
*K. pneumoniae+M. catarrhalis*	1	0	1	
*K. pneumoniae*	1	0	1	
*A. Pittii*	1	0	1	
***Aspergillus***	**1**	**0**	**1**	0.306

*ADV* Adenovirus, *HBoV* Human bocavirus, *RSV* Respiratory syncytial virus, *PIV* Human parainfluenza virus, *HRV* human rhinovirus, *HMPV* human metapneumovirus, *M. pneumoniae Mycoplasma pneumoniae*, *S. pneumoniae Streptococcus pneumoniae*, *H. influenzae Haemophilus influenz*ae, *S. aureus Staphylococcus aureus, M. catarrhalis Moraxella catarrhalis, P. aeruginosa Pseudomonas aeruginosa, K. pneumoniae Klebsiella pneumoniae, MTB Mycobacterium tuberculosis, A. pittii Acinetobacter pittii*

### Multiple logistic regression analysis for SMPP markers

To evaluate markers for differentiating SMPP and GMPP, 417 cases were subjected into a non-conditional multiple logistic regression analysis of. The level of IL-2 was a protective factor, and white blood cell count, CRP, IL-5, IL-6, fever duration, and digestive system complications had significantly higher predictive values as risk factors for SMPP, and the corresponding odds ratio values were 1.125, 1.047, 1.485, 1.016, 1.286, and 4.900, respectively (Table 4).

**Table 4:** Logistic regression analysis for the related factors predicting the SMPP

**Positive Variables**	**OR**	**95% CI**		***P* value**
		**Lower**	**Upper**	
Gastrointestinal involvement	4.900	1.251	19.190	0.023
Fever duration	1.286	1.113	1.486	0.001
WBC	1.125	1.019	1.243	0.020
CRP	1.047	1.013	1.083	0.007
IL-2	0.904	0.821	0.996	0.041
IL-5	1.485	1.150	1.918	0.002
IL-6	1.016	1.000	1.033	0.048

Abbreviations: *CI* Confidence interval, *OR* Odds ratio, *WBC* White blood cell, *CRP* C-reactive protein, *IL* Interleukin

## Discussion

Although *M. pneumoniae* often causes community-acquired pneumonia, the epidemiology of severe infections that pose a life-threatening risk in children is poorly characterized [7]. Here, we explored the severity of MPP and its related factors in children with MPP from January and December 2022 and present the following key findings: 1) half of the MPP cases were severe and occurred mainly in winter, 2) a greater proportion of children with SMPP received antibiotics other than macrolides, and 3) higher levels of pro-inflammatory ILs, gastrointestinal complications as well as duration of fever were risk factors for severe MPP.

MPP can cause severe pulmonary infections and extrapulmonary complications in adults and children [11]. During the 2010–2013 *M. pneumoniae* epidemic, the hospital mortality rate of adult patients with MPP admitted to intensive care was 29.4% [3]. A large study of paediatric patients with MPP from 2011–2019 in Taiwan revealed emerging cases requiring extracorporeal membrane oxygenation [12]. Carrim et al. treated 103 hospitalised patients with MPP and found that an age of <6 years was an independent risk factor of severe pneumonia [13]. In this study, we observed a severe rate of more than half of hospitalised children with MPP, and extrapulmonary complications included digestive, cardiovascular, and neurological system complications. A series of 332 paediatric patients hospitalised with MPP between 2007–2017 revealed that 25.6% (85/332) of the children experience complications related to the skin, digestion, nervous system, and cardiovascular system [14]. A study conducted in China found that 55.9% (33/59) of children with MPP between 2013-2014 had myocardial damage [15]. In our study, myocardial damage, pericardial effusion, and heart failure were observed especially in the SMPP group. Kammer et al. showed that 24.7% (22/89) of paediatric patients hospitalised in Germany between 2000-2013 had neurological symptoms and signs [16]. We observed febrile seizures, epilepsy, and encephalitis only in the SMPP group. Mia et al. reported that in 2010–2011, 33% (246/746) of children with MPP admitted to a paediatric hospital in Denmark had nausea or vomiting complications [17]. Othman et al. reported children with MPP exhibited coryza, diarrhoea and vomiting especially in children less than 5 years[18]. We observed that the gastrointestinal complication was a dependent risk factor for SMPP. These data suggest that extrapulmonary complications of MPP are common in children and are more frequent in severe cases. Therefore, the management of extrapulmonary complications should be a critical part of the therapeutic regimens for MPP.

In this report, elevated leukocyte levels with a predominance of neutrophils were observed in children with severe pneumonia. Shimizu et al. reported that after *M. pneumoniae* infection, the *M. pneumoniae* lipid-associated membrane proteins can activate the toll-like receptor, leading to an increase in neutrophils [19]. CRP and PCT are sensitive indicators of the acute phase of inflammation. They are useful in identifying severe disease caused by MPP [20]. CRP levels are correlated with infection severity [21]. In the present study, patients with SMPP had a lower percentage of NK cell abnormalities than those with GMPP. Genome pathway analysis revealed that the NK cell-mediated cytotoxicity pathway was significantly upregulated in children with MPP [22]. Another study found that the number of NK cells and the expression of CD158b on their surface were altered in patients with severe acute respiratory syndrome and correlated with disease severity [23]. The study found that patients with severe disease had a significantly lower percentage of NK cells compared to those with GMPP. This suggests that NK cells may serve as a marker of disease severity; however, further mechanistic studies are required.

Cytokines are widely used as markers in children with infectious diseases [24]. In the present study, we observed the elevated serum concentrations of INF-γ, IL-2, IL-5, IL-6, IL-8, and IL-10 in patients with SMPP. ILs are a class of cytokines produced by a various of cells that play important regulatory roles in the immune system and have been implicated in the development and progression of MPP [25–27]. In severe pneumonia, immune cells in the lungs (e.g., macrophages and neutrophils) release interleukins, leading to an enhanced inflammatory response. *M. pneumoniae* infection increases IL-6 gene expression and its protein secretion [25]. Serum concentration of IL-6 could potentially indicate the severity and outcomes of MPP [26]. Our results were similar to those of Zhang et al., with the levels of IL-2, IL-10 and IFN-γ being higher in the SMPP group than in the GMPP group (*p*<.05) [27]. IFN-γ is one of the major cytokines involved in the inflammatory response after *M. pneumoniae* infection and can increase macrophage lysosomal activity and stimulate macrophage secretion of pro-inflammatory factors to exacerbate the inflammatory response [28]. Esposito et al. discovered that IL-5 was the only cytokine that presented substantially differences in the serum of children with acute *M. pneumoniae* infection and wheezing [29]. Narita et al. found that in patients with pleural effusion and a persistent fibrotic change in the lungs caused by *M. pneumoniae* infection, IL-8 was also detected. This indicates the crucial role for IL-8 in the pathogenesis of MPP [30]. It is important to note that the relationship between ILs and severe pneumonia is complex, and that IL levels and actions may be influenced by individual differences, infectious agents, and host immune status. Current strategies for the treatment of severe pneumonia focus on anti-infective therapy and a combination of supportive therapeutic measures [31]. To regulate the inflammatory response, several anti-inflammatory and immunomodulatory drugs need to be investigated for clinical application.

Previous studies have described bacterial co-infection in severely ill children with MPP [32]. In a study aimed at evaluating the frequency and impact of bacterial co-infection in children hospitalised with MPP, Song et al. showed that 2% (173/8612) of children with MPP had bacterial co-infection between 2006–2014, half of which were with *S. pneumoniae* [33]. In another study, 15.3% (9/59) of hospitalized children with MPP in Korea were found to have bacterial co-infection. In children under the age of 5 years, co-infection with *S. pneumoniae* was more likely to result in a longer duration of fever and hospital stay, compared to those infected with *M. pneumoniae* alone [34]. In addition, reports suggest that viruses, such as rhinovirus, may be independent causative agents of pneumonia, including severe pneumonia [35]. Human rhinovirus has emerged as an important cause of pneumonia due to its severity and poor prognosis in adults and children [32, 36]. We observed a substantially increase in the rate of viral and bacterial co-infections in children with SMPP. The results imply that early identification and swift, efficient antibiotic or antiviral treatment in SMPP children is crucial for preventing disease progression. Further investigation is warranted to determine the pathogenic role of this virus and identify specific bacteria that may be the culprit for the severe clinical presentations.

Our study has several limitations. The first one is that it was a retrospective study conducted in a single centre. Second, only inpatients were included, which restricts the generalizability of the results to other patients, particularly those with mild MPP who did not require hospitalization.

## Conclusions

Conclusively, a much higher proportion of SMPP children are prone to extrapulmonary complications and viral or bacterial co-infections. Children with severe illness present with elevated inflammation and decreased adaptive immunity. Consequently, hormonal, prophylactic and anticoagulant therapies, as well as the use of antibiotics other than macrolides for bacterial co-infections, could be incorporated into the treatment regimens for the effective management of children with SMPP.

## Data Availability

Data is provided within the Figshare repository (https://doi.org/10.6084/m9.figshare.25339039.v1).

## Abbreviations

MP: *Mycoplasma pneumoniae*
MPP: *Mycoplasma pneumoniae* pneumonia
SMPP: Severe *Mycoplasma pneumoniae* pneumonia
GMPP: General *Mycoplasma pneumoniae* pneumonia
CHH: Children’s Hospital of Hebei
BALF: Bronchoalveolar lavage fluid
IS: Induced sputum
CRP: C-reactive protein
PCT: Procalcitonin
IL: Interleukin
NK: Natural killer
RSV: Respiratory syncytial virus
PIV: Human parainfluenza virus
HCoV: Human coronavirus
ADV: Adenovirus
HBoV: Human bocavirus
HMPV: Human metapneumovirus
*H. influenzae*: *Haemophilus influenza*
*K. pneumonia*: *Klebsiella pneumoniae*
*M. catarrhalis*: *Moraxella catarrhalis*
*MTB*: *Mycobacterium tuberculosis*
*S. pneumoniae*: *Streptococcus pneumoniae*
*S. aureus*: *Staphylococcus aureus*
*S. capitis*: *Staphylococcus capitis*
*A. pittii*: *Acinetobacter pittii*
